# Delayed Tibial Osteomyelitis after Anterior Cruciate Ligament Reconstruction with Hamstrings Autograft and Bioabsorbable Interference Screw: A Case Report and Review of the Literature

**DOI:** 10.1155/2017/6383526

**Published:** 2017-10-15

**Authors:** Kevin S. Weiss, Justin M. Weatherall, Jen Eick, James R. Ross

**Affiliations:** ^1^Broward Health Medical Center, Nova Southeastern University College of Osteopathic Medicine, Fort Lauderdale, FL 33316, USA; ^2^Boca Care Orthopedics, Florida Atlantic University College of Medicine, Boca Raton, FL 33431, USA; ^3^Lynn University Athletic Department, 3601 N. Military Trail, Boca Raton, FL 33431, USA

## Abstract

Osteomyelitis following arthroscopically assisted anterior cruciate ligament (ACL) reconstruction has rarely been reported in the literature. We report a case of a 20-year-old female who had delayed tibial osteomyelitis and a pretibial cyst with culture-positive, oxacillin sensitive* Staphylococcus epidermidis* 15 months after an ACL reconstruction with hamstring autograft. Soft tissue fixation within the tibial tunnel was with a poly-L-D-lactic acid (PLDLA) bioabsorbable interference screw. The patient underwent surgical treatment with curettage, debridement, hardware removal, and bone grafting of the tibial tunnel followed by a course of intravenous antibiotics. Arthroscopic evaluation demonstrated an intact ACL graft without any evidence of intra-articular infection. The patient returned to collegiate athletics without any complications. While the most common biologic complications include pretibial cysts, granuloma formation, tunnel widening, and inflammatory reactions, infection is exceedingly rare. Late infection and osteomyelitis are also rare but can occur and should be considered in the differential diagnosis.

## 1. Introduction

Postoperative infection is a rare, however, challenging complication after arthroscopically assisted anterior cruciate ligament (ACL) reconstruction. The incidence of infection after ACL reconstruction has been reported to range from 0.1% to 1.7% among retrospective studies [1–8]. Osteomyelitis following ACL reconstruction has been even more rarely reported in the literature [9–12]. Prompt diagnosis and treatment with debridement of the infectious material and a bacterial-specific antibiotic regimen are critical for eradication. However, early diagnosis may be challenging, as other inflammatory processes, in the setting of a bioabsorbable screw, may mimic an underlying infection.

Biodegradable interference screws have become more common in ACL reconstruction surgery. The mechanical and degradable properties of biodegradable screws make them an excellent alternative for fixation given the complications associated with metallic interference screws such as artifact with MRI imaging, graft damage or rupture, and need for removal during revision surgery [13–16]. Various types of polymers are used in the composition of biodegradable screws. The degradation properties of biodegradable screws depend on the polymer used. Most incorporate poly-L-lactic acid (PLLA), polyglycolic acid (PGA), copolymers with PLLA and PGA, or polylactide carbonate (PLC) which is poly-D,L-lactide (PDLLA) combined with calcium carbonate, an osteoconductive agent [13, 17–21]. Numerous complications such as cyst formation, tunnel widening, abscess formation, inflammatory reactions, pretibial cyst formation, granuloma formation, screw breakage, and implant migration have been reported for biodegradable implants [13, 22–29]. We present a case of increasing pain and swelling, 15 months after an ACL reconstruction with hamstring autograft using a poly-L-D-lactic acid (PLDLA) bioabsorbable interference screw.

## 2. Case Report

A 20-year-old female presented to our clinics approximately 15 months after undergoing an arthroscopically assisted ACL reconstruction using hamstring autograft by another orthopedic surgeon. Details regarding the surgery and perioperative and postoperative care were obtained from hospital records. Graft fixation was performed using suspensory fixation (ToggleLoc, Biomet, Warsaw, Indiana) on the femoral side and a 9.0 mm × 30 mm bioabsorbable interference screw (ComposiTCP, Warsaw, Indiana) on the tibial side. This screw has a composite blend of 40% poly-L-D-lactic acid (PLDLA) and 60% beta-tricalcium phosphate. Intravenous cefazolin was administered for perioperative antibiotic prophylaxis. The postoperative course was otherwise routine, without any wound complications or difficulties with physical therapy progression. The patient ultimately returned back to soccer at approximately 9 months, after completing an appropriate ACL return to play program.

**Figure 1:**
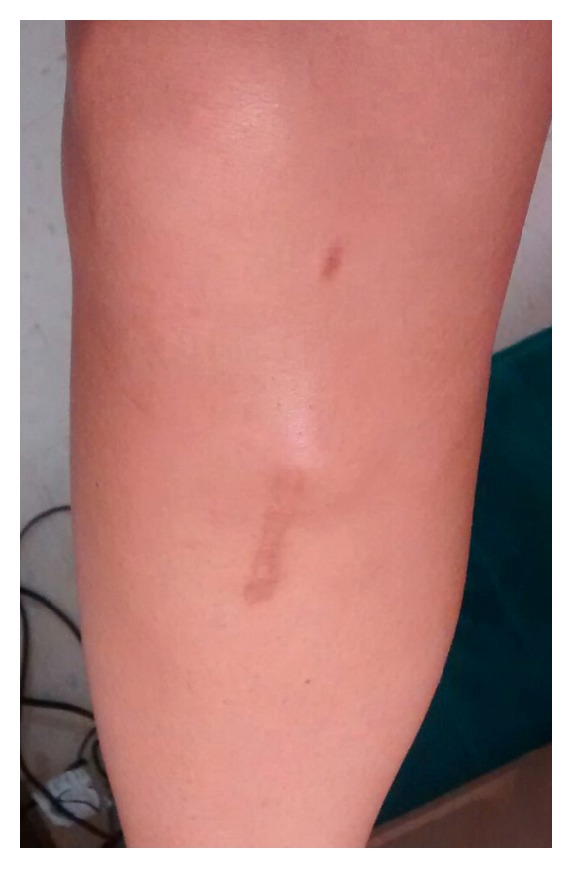
Clinical photos of the pretibial swelling and mass that is noted at the proximal aspect of the incision.

The patient began to notice localized swelling in the proximal medial tibia at the proximal end of the prior incision (Figure 1). On examination, the patient had full symmetric range of motion and no effusion. Lachman's testing was 1A with no increased laxity to varus and valgus stress at 0° and 30° of flexion compared to the contralateral knee. The surgical incision over the proximal medial tibial was healed; however, there was a focal area of nodular swelling at proximal aspect of the incision that measured approximately 3 cm × 3 cm. There was slight pretibial edema surrounding this region, minimal increase in warmth, and no erythema. The patient reported no recent history of fevers or chills.

**Figure 2:**
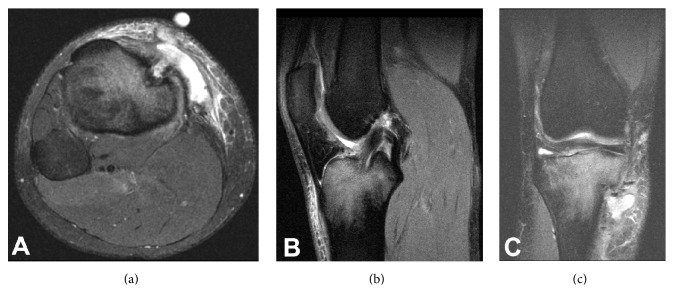
(a) Axial slice of the MRI demonstrating the pretibial fluid collection in continuity with the orifice of the tibial tunnel. (b) T2 sagittal image demonstrates an intact ACL graft with adjacent tibial bone marrow edema. (c) T2 coronal image again demonstrates the pretibial fluid collection in continuity with the tibial tunnel and proximal tibial edema.

Laboratory examination of her peripheral blood demonstrated a white blood cell (WBC) count of 6.7 × 10^3^/UL (normal, 4–10 × 10^3^/UL) with 62% granulocytes. Erythrocyte sedimentation rate (ESR) measured 6 mm/hr (normal, 0–30 mm/hr) and C-Reactive Protein (CRP) measured <0.29 mg/dL (normal, <0.80 mg/dL). Magnetic resonance imaging (MRI) of the knee demonstrated a complex soft tissue fluid collection in the anterior aspect of the tibia, in continuity with the tibial tunnel. Additionally, there was bone marrow edema of the proximal tibia (Figure 2). Based on these findings in conjunction with the physical examination, we suspected her symptoms to be a result of a reaction to the bioabsorbable screw; however, we could not exclude infection as an underlying cause. Due to the patient's continued symptoms, we recommended a diagnostic arthroscopy, pretibial cyst removal, irrigation and debridement, removal of hardware, and bone grafting of the tibial tunnel.

**Figure 3:**
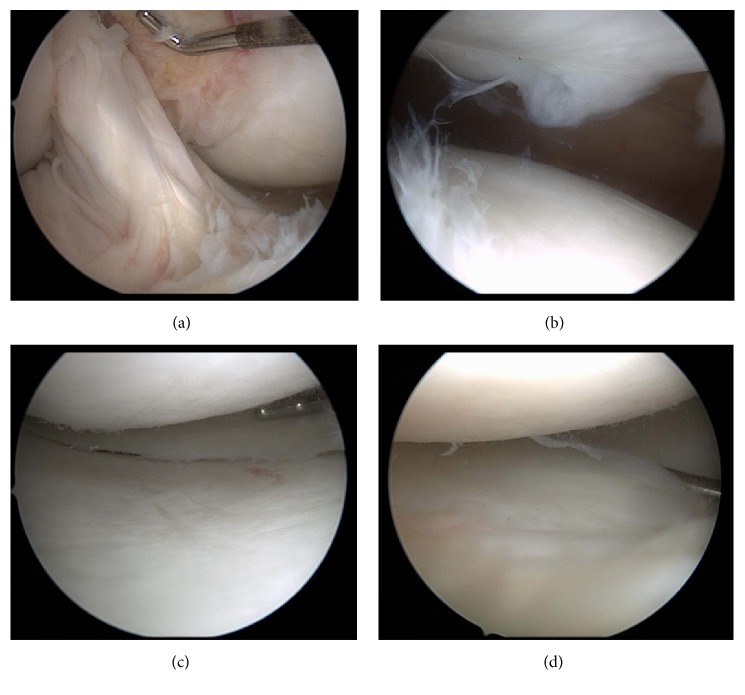
(a) Intact ACL graft. (b) Grade 2 chondral changes in the medial face of the patella. (c) Intact articular cartilage of the medial tibial plateau, no tearing in the medial meniscus. (d) Grade 1 and 2 changes on the central aspect of the lateral tibia plateau.

The diagnostic arthroscopy demonstrated International Cartilage Repair Society (ICRS) grade 2 chondral changes in the medial facet of the patella, which was treated with chondroplasty. The posterior cruciate ligament (PCL) and the ACL reconstruction were intact. The medial meniscus was normal. Slight grade 1 changes of the medial femoral condyle were noted. The articular cartilage of the medial tibial plateau was normal. Grade 1 and 2 changes on the central aspect of the lateral tibia plateau were noted. The lateral femoral condyle cartilage was normal (Figure 3). There was a small amount of fibrous scar tissue anterior to the ACL graft, which was debrided. There were no signs of infection or inflammation, and the synovial tissue was not inflamed.

**Figure 4:**
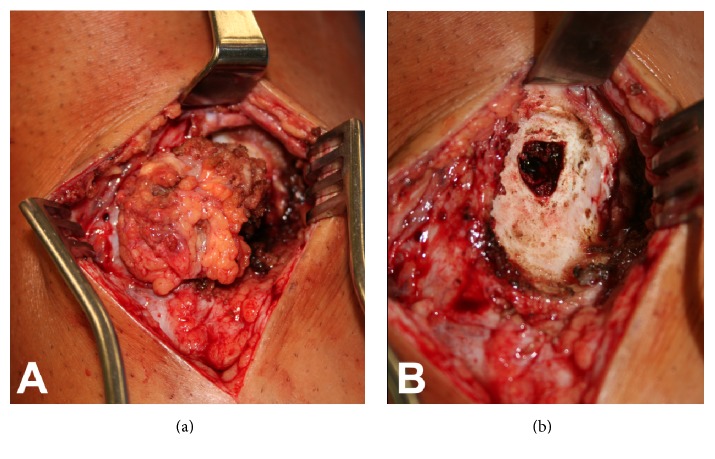
(a) The pretibial soft tissue mass demonstrates direct communication with the tibial tunnel. (b) The tibial tunnel is well visualized after removal of the soft tissue mass. There is a lack of soft tissue deep to the skin, which prompted the sartorial fascia transfer over the exposed bone.

To address the tibial cyst, the prior surgical incision was used. The underlying soft tissue mass was visible after making the skin incision. The mass was found to communicate with the tibial tunnel (Figure 4(a)). The soft tissue mass was resected en bloc and sent for pathology and routine aerobic culture, anaerobic culture, acid fast, and fungal cultures were sent. No hardware was present in the tibial tunnel; however, there was fibrous tissue and a chalky white substance, likely from breakdown of the interference screw. This tissue was removed and sent for culture. There was also no bony incorporation of the interference screw; however there was good integration of the ACL graft to the tunnel walls and the proximal tibial tunnel. There was no communication of the tibial tunnel with the joint. This tissue was removed and sent for culture. A motorized burr was used to freshen the tunnel and thoroughly debride the remaining tunnel wall, to expose cancellous bone. A sample of cancellous bone was then harvested with a curette and sent for culture. The incision and tunnel were thoroughly irrigated, and allograft cancellous chips were placed to fill the tunnel voids. Given the soft tissue defect in the area of the tibial tunnel (Figure 4(b)) the sartorial fascia was elevated and transferred proximally to cover the tibial tunnel to provide additional soft tissue coverage under the skin incision. The patient was provisionally started on oral cephalexin.

**Figure 5:**
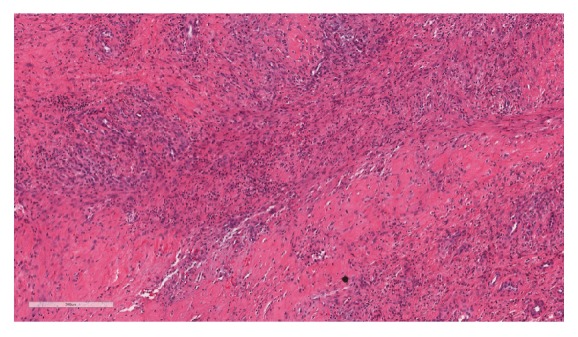
Representative low power view of sampled fibrous tissue with acute and chronic inflammation.

The aerobic intraoperative cultures grew oxacillin sensitive* Staphylococcus epidermidis*. Surgical pathology of the cyst demonstrated benign fibroadipose tissue with acute and chronic inflammation, fibrosis, and multinucleated giant cell reaction and associated histocytic proliferation. Surgical pathology of the tibial bone demonstrated acute and chronic inflammation, fibrosis, and dystrophic calcifications, consistent with osteomyelitis. Pathologic specimen from the tibial tunnel is shown in Figure 5. Consultation with an infectious disease specialist was obtained, and the patient was diagnosed with osteomyelitis and was treated with a 5-week course of intravenous ceftriaxone. Due to elevated liver enzymes, the patient was switched to intravenous daptomycin for an additional one week, for a total of six weeks of intravenous antibiotics. Final laboratory examination of the patient's peripheral blood demonstrated a white blood cell (WBC) count 5.5 × 10^3^/UL (normal, 4–10 × 10^3^/UL) with 62% granulocytes.

**Figure 6:**
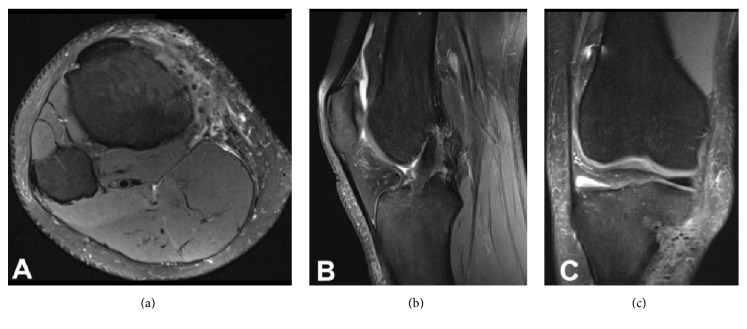
(a) T2 axial slice of the MRI demonstrating resolution of the pretibial fluid collection. (b) T2 sagittal image demonstrates an intact ACL graft resolution of tibial bone marrow edema. (c) T2 coronal image again demonstrates resolution of pretibial fluid collection and proximal tibial edema.

The patient underwent a strengthening and rehabilitation program and ultimately returned back to collegiate soccer without any recurrence of pain, swelling, or signs of infection. The patient has not experienced any symptoms of instability. Follow-up period was 4 months. An MRI at the 4-month time period of her right tibia is shown in Figure 6 and demonstrated clearance of the infection.

## 3. Discussion

Infection after ACL reconstruction is a rare complication. The average interval between surgery and the presentation of joint infection symptoms ranges from 1 to 3 weeks. Osteomyelitis secondary to ACL reconstruction is an even more rare complication. To our knowledge, this is the first reported case of post-ACL osteomyelitis caused by* Staphylococcus epidermidis*, which was limited to the tibia without any involvement of the knee joint.

*Staphylococcus epidermidis* is a coagulase-negative bacteria that constitutes 48% of coagulase-negative bacterial infections [30].* S. epidermidis* and* S. aureus* are the most prevalent species encountered in orthopedic device related infections [31, 32]. The prevalence of* S. epidermidis *approaches 50% of late-developing orthopedic related infections which is attributed to the lack of virulence factors compared to other bacteria that are commonly found in acute infections [33]. The ability to rapidly form a biofilm may be the most critical factor possessed by* S. epidermidis*; however this varies among the* S. epidermidis* strains [34, 35]. Of the coagulase-negative staphylococci involved in bone and joint infections,* S. epidermidis* is the major isolate in 81 percent of the cases [36].

A recent study of over 10,000 ACL reconstructions demonstrated that hamstring tendon autografts had a significantly higher rate (8.24 times higher) of postoperative infection (0.61%) compared to patellar tendon autograft (0.07%) [37]. Thirty-five percent (7/20) of the hamstring graft infections were due to coagulase-negative* Staphylococcus* species. A recent study of 60 patients undergoing hamstring autograft ACL had a portion of their excess tendon sent for culture, which demonstrated a 16.7% positive culture growth, despite the absence of any clinical infections. Forty-percent (4/10) of these positive cultures were due to* S. epidermidis* [38]. This has been attributed to increased handling time, surgical gloves, and surgical instrumentation; however, no definite cause is currently known.

The signs and symptoms of a late infection present a challenge in the setting of a bioabsorbable interference screw. Biologic reactions to these screws have been shown to include pretibial cysts, granuloma formation, tunnel widening, and inflammatory reactions [13, 14, 26, 39–41]. Several other complications have been documented in the literature, which include screw breakage, fixation failure, screw migration, osteolysis, and joint effusions [14]. Pretibial cysts due to bioabsorbable interference screws have been reported to be most likely due to an inflammatory reaction caused by the breakdown of the biodegradable screws. One case report demonstrated tibial tunnel osteolysis with granuloma formation 6 months after an ACL reconstruction with hamstrings autograft using a polylactide carbonate (PLC) interference screw [13]. A retrospective review of 7 patients with pretibial cysts secondary to breakdown of PLLA interference screws was reviewed and demonstrated cystic reaction due to fragmented particles from the screw [39]. A prospective review of 59 patients that underwent ACL reconstruction with PLC interference screws demonstrated multiple adverse reactions (39%), including pretibial swelling (34%) and synovitis (15%) [40]. These reactions may present early or late after surgery. Umar et al. described the onset of pretibial swelling 30 months after ACL reconstruction with PLLA interference screw. Surgical exploration demonstrated chalky debris within the tibial tunnel and histologic evaluation showed an aseptic foreign body reaction with multinucleated giant cells [41].

While infection can present as a complication in any orthopedic surgery, to our knowledge, there are no documented cases of delayed osteomyelitis with* S. epidermidis* in the setting of bioabsorbable interference screw fixation. Furthermore, infection is rarely documented in patients with tibial cysts or swelling after ACL reconstruction with bioabsorbable screws. In a retrospective review by Ramsingh et al., 5% of patients (14/273) presented with pretibial pain and swelling at a mean of 16 months after ACL reconstructions using various bioabsorbable screws for tibial fixation. No evidence of infection was found in any of the intraoperative specimen cultures [42]. However, these aseptic cases may not have underwent appropriate culture evaluation, as a recent case series of 2 patients demonstrated an infection with* Propionibacterium acnes* when cultured anaerobically for 10 days on thioglycolate broth [43].

## 4. Conclusion

Bioabsorbable screws have become a common alternative for interference fixation during ACL reconstruction. While the most common biologic complications include pretibial cysts, granuloma formation, tunnel widening, and inflammatory reactions, infection is exceedingly rare. Late infection and osteomyelitis are also rare but can occur. Infection should be considered in the differential diagnosis of a patient presenting with localized knee pain, swelling, and normal laboratory analysis, which may otherwise be attributed to a biologic reaction from a bioabsorbable interference screw. Prompt diagnosis with surgical debridement and intravenous antibiotics may allow for eradication and a good clinical outcome.
